# immuneSIM: tunable multi-feature simulation of B- and T-cell receptor repertoires for immunoinformatics benchmarking

**DOI:** 10.1093/bioinformatics/btaa158

**Published:** 2020-04-14

**Authors:** Cédric R Weber, Rahmad Akbar, Alexander Yermanos, Milena Pavlović, Igor Snapkov, Geir K Sandve, Sai T Reddy, Victor Greiff

**Affiliations:** b1 Department of Biosystems Science and Engineering, ETH Zürich, 4058 Basel, Switzerland; b2 Department of Immunology, University of Oslo, 0372 Oslo, Norway; b3 Department of Informatics, University of Oslo, 0373 Oslo, Norway

## Abstract

**Summary:**

B- and T-cell receptor repertoires of the adaptive immune system have become a key target for diagnostics and therapeutics research. Consequently, there is a rapidly growing number of bioinformatics tools for immune repertoire analysis. Benchmarking of such tools is crucial for ensuring reproducible and generalizable computational analyses. Currently, however, it remains challenging to create standardized ground truth immune receptor repertoires for immunoinformatics tool benchmarking. Therefore, we developed immuneSIM, an R package that allows the simulation of native-like and aberrant synthetic full-length variable region immune receptor sequences by tuning the following immune receptor features: (i) species and chain type (BCR, TCR, single and paired), (ii) germline gene usage, (iii) occurrence of insertions and deletions, (iv) clonal abundance, (v) somatic hypermutation and (vi) sequence motifs. Each simulated sequence is annotated by the complete set of simulation events that contributed to its *in silico* generation. immuneSIM permits the benchmarking of key computational tools for immune receptor analysis, such as germline gene annotation, diversity and overlap estimation, sequence similarity, network architecture, clustering analysis and machine learning methods for motif detection.

**Availability and implementation:**

The package is available via https://github.com/GreiffLab/immuneSIM and on CRAN at https://cran.r-project.org/web/packages/immuneSIM. The documentation is hosted at https://immuneSIM.readthedocs.io.

**Contact:**

sai.reddy@ethz.ch or victor.greiff@medisin.uio.no

**Supplementary information:**

Supplementary data are available at *Bioinformatics* online.

## 1 Introduction

**Fig. 1.**
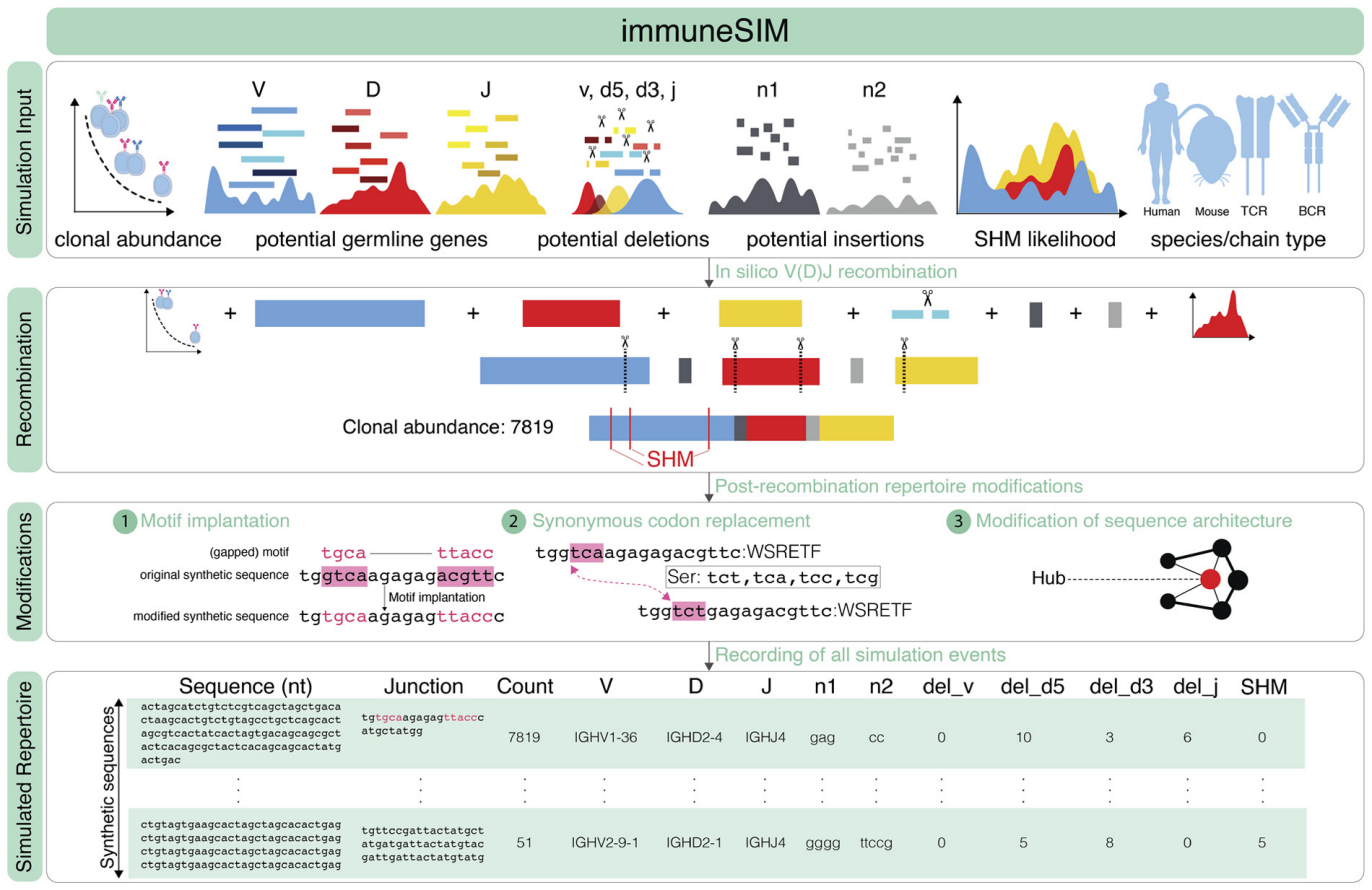
immuneSIM simulates fully (single, paired) annotated tunable immune repertoires that are either highly similar (native-like) or deviating (aberrant, see main text for definition) from experimental immune repertoires. All major immune repertoire features, such as clonal abundance, germline genes, deletions and insertions and somatic hypermutation, are tunable. Post *in silico* recombination, the immuneSIM-generated immune receptor repertoires may be further modified by (i) implantation of motifs, (ii) codon replacement and (iii) change of sequence similarity architecture

Targeted deep sequencing of adaptive immune receptor repertoires (AIRR-seq data, Breden *et al.*, 2017) has become a key resource for immunodiagnostics and immunotherapeutics research. Consequently, there exists a rapidly growing number of immune receptor informatics tools for germline gene annotation, diversity and overlap estimation, network architecture (sequence similarity) and machine learning analysis (Brown *et al.*, 2019; Yaari and Kleinstein, 2015). To benchmark and assess the performance of these tools, synthetic ground truth immune receptor datasets with complete information on all repertoire feature dimensions investigated or used in these tools (e.g. germline gene usage, insertion and deletions, and clonal abundance; Fig. 1) are required (Brown *et al.*, 2019; Yaari and Kleinstein, 2015). Therefore, there is a need for a computational framework that enables the simulation of native-like immune receptor repertoires as well as repertoires that differ in single- or multiple feature dimensions while simultaneously allowing for the tracing of all immunologically relevant simulation parameters. To address this gap in the landscape of immune receptor simulation tools that are focused predominantly on generating native-like repertoires (Marcou *et al.*, 2018; Safonova *et al.*, 2015; Yermanos *et al.*, 2017), we here present the immuneSIM R package, which allows the tunable multi-feature simulation of human and mouse BCR and TCR repertoires (single-chain and paired full-length variable regions) with traceable simulation event-level annotation for each of the simulated sequences.

The user has full control over the following immunological features: V-, D-, J-germline gene set and usage, occurrence of insertions and deletions, clonal sequence abundance and somatic hypermutation. Post-sequence simulation, the generated immune receptor sequences may be further altered by the addition of custom sequence motifs, synonymous codon replacement as well as the modification of the sequence similarity architecture (Fig. 1). We validated that immuneSIM can generate immune repertoires that are similar to experimental repertoires (native-like) by evaluating a range of repertoire similarity measures. immuneSIM can also generate aberrant immune receptor repertoires to replicate a broad range of experimental, immunological or disease settings (Arora *et al.*, 2019; Brown *et al.*, 2019) (Supplementary Figs S2–S7).

## 2 Package description

immuneSIM enables the simulation of native-like and aberrant repertoires for the benchmarking of immunoinformatics tools. In order to simulate immune receptor repertoires that mimic native-like repertoires, immuneSIM contains reference experimental data from human and mouse studies (Supplementary Table S1). ImmuneSIM allows for further customization by permitting the inclusion of alternative experimental or user-created reference datasets as well as the simulation of *aberrant* repertoires with feature distributions different from those observed in the input experimental parameters provided by the immuneSIM package. The *in silico* recombination process (Fig. 1 and Supplementary Fig. S1) starts by sampling V-, D- and J-genes according to a given frequency distribution (possibly sampled from input datasets), followed by the simulation of deletion events for the V- and D-genes. To increase the probability of providing the user with in-frame junctional regions, the J-gene deletion length is chosen in such a way that the J-gene anchor (i.e. the nucleotide pattern that marks the J region of the CDR3) (Giudicelli and Lefranc, 2011) remains in-frame. Likewise, the n1 (5′ of D-gene) and n2 (3′ of D-gene) insertion sequences are sampled from a subset of observed insertion sequences to ensure the maximal probability of generating an in-frame sequence. Following the assembly of the V, n1, D, n2 and J fragments into a full V(D)J sequence, a clone abundance is assigned to it, and somatic hypermutation (for B-cell receptors only) based on the R package AbSim (Yermanos *et al.*, 2017) may be applied. Depending on the V-, (D-), J-genes sampled, it is possible that the simulated sequences contain stop codons; any such unproductive sequences are automatically discarded. immuneSIM continues to simulate recombined sequences until the user-defined number of sequences has been reached. To reach very large number of diverse BCR and TCR repertoires, simulations may be parallelized (Supplementary Figs S8 and S9). Each immuneSIM-generated sequence is annotated with all simulation events that led to its generation including species (human, mouse), chain type (single, paired; TCRβ/TCRα, IgH/Igκ, Igλ), clonal abundance, V-, (D-), J-germline gene usage, information on deletions, insertions (n1, n2) and junctional recombination (complementarity determining region 3, CDR3). Additionally, modifications such as synonymous codon replacement (relevant for testing the analytical performance of nucleotide and amino acid sequence-based methods) and alteration of repertoire sequence similarity architecture (relevant for testing graph-based tools) can be performed (Fig. 1). Furthermore, a flexible motif implantation function allows the user to simulate specific sequence motifs through the controlled insertion of short sequence motifs of various complexities (k-mers of various sequence lengths and diversity at specified frequencies). Sequence motifs have been previously shown to be implicated in the prediction of public and private clones (Greiff *et al.*, 2017) as well as antigen binding and disease course (Dash *et al.*, 2017; Glanville *et al.*, 2017).

## 3 Validation: simulating native-like and aberrant immune receptor repertoires

To validate the similarity between simulated and experimental repertoires, we simulated repertoires using standard settings based on experimental data and aberrant repertoires with parameters introducing noise in various dimensions (Supplementary Table S2). We first compared simulated BCR and TCR repertoires to experimental datasets in terms of CDR3 length distribution, VDJ usage, positional amino acid frequencies and k-mer co-occurrence (gapped subsequence structures) (Supplementary Figs S2–S5). Briefly, we validated that, for example, murine IgH repertoires simulated with the standard parameters replicated V-, D- and J-gene frequencies between input and output (*r*_Spearman_ ≥ 0.985), whereas aberrant simulated BCR and TCR repertoires showed larger deviations (*r*_Spearman_ ≥ 0.8). Similarly, the amino acid frequency distribution differed only slightly compared to the naïve repertoire when using standard parameters (mmse across positions = 0.000486) in contrast to aberrant repertoires that were more distant (mmse = 0.001659). Finally, gapped-k-mer subsequence usage correlated highly between standard simulations and experimental repertoires (*r*_Spearman_ = 0.86, at *k* = 3, *m* ≤ 3, where *k* is the k-mer amino acid length and *m* is the number of amino acid gaps) while aberrant repertoires showed more distinct gapped-k-mer patterns (*r*_Spearman_ = 0.74). To further substantiate the congruence of experimental and immuneSIM generated repertoires, we determined the extent to which the internal annotation of simulated repertoires overlapped with IMGT’s HighV-Quest, a commonly used annotation tool (Supplementary Figs S6 and S7). We found up to 99% of simulated sequences were annotated as ‘productive’ and ‘in-frame’ by IMGT HighV-Quest. Among these sequences, 94% of the time the junction identified by immuneSIM was found to be identical to that of IMGT. The V and J annotation overlapped in >97% of simulated sequences, while D annotations, a generally more difficult problem due to deletions and insertions, showed an overlap of ∼60%. Taken together, these results support the notion that immuneSIM repertoires are nearly indistinguishable from experimental repertoires with respect to major statistical descriptors and thus can serve as a reliable basis for benchmarking immunoinformatics tools. Finally, immuneSIM may serve for tool stress-testing analysis, for example benchmarking machine learning methods (Emerson *et al.*, 2017; Greiff *et al.*, 2017), using implanted sequence motifs at various frequencies and complexities.

## Funding

This work was funded by the Swiss National Science Foundation (Project no. 31003A_170110 to S.T.R.).

*Conflict of Interest*: none declared.

## Supplementary Material

btaa158_Supplementary_Data
