# Supplementary material for: immuneSIM: tunable multi-feature simulation of B- and T-cell receptor repertoires for immunoinformatics benchmarking
Source: Bioinformatics. 2020 Apr 14;36(11):3594–6. doi: 10.1093/bioinformatics/btaa158 (PMC7334888; doi:10.1093/bioinformatics/btaa158)
Supplement: btaa158_Supplementary_Data [file btaa158_supplementary_data.zip › btaa158-Suppl_Data/05_immuneSIM_Supplementary_Data_Figures.pdf]

Supplementary Figure 1

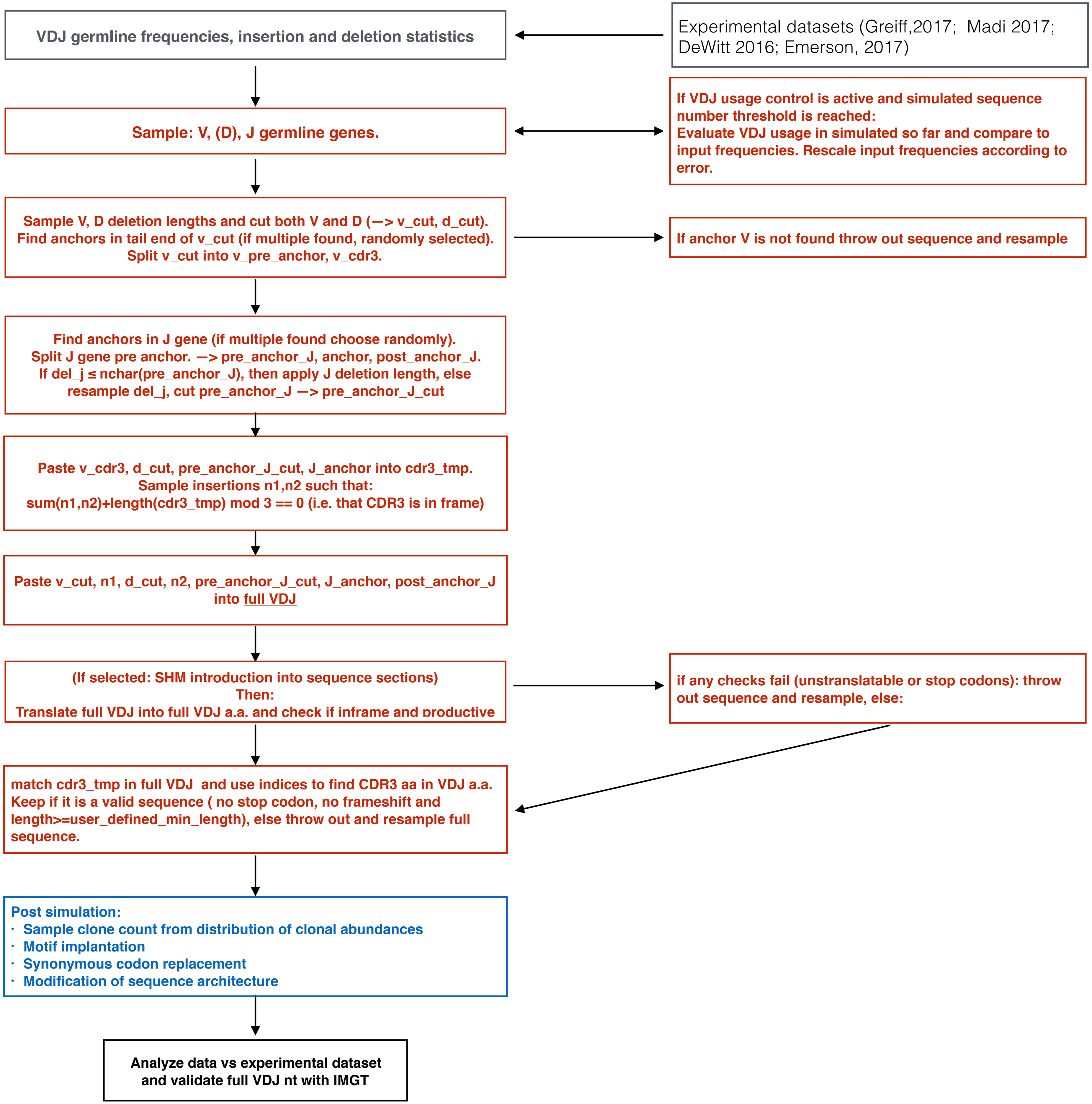

SFig. 1 Flowchart of immune receptor simulation in the R package immuneSIM.
immuneSIM allows simulation of immune repertoires via an in silico V(D)J recombination algorithm with input parameters (VDJ frequencies, insertion sequences and deletion lengths) in a user-defined fashion based on experimental data. The germline V, D and J genes are sampled according to usage frequencies defined in the input. After germline gene sampling, a CDR3 anchor is identified in the V and J gene. Subsequently, V and D and J deletion lengths are sampled randomly. Finally, the n1 and n2 insertions are sampled from a subset of insertions such that their length complements the current preliminary CDR3 resulting in an in-frame CDR3. The resulting nucleotide sequence is subsequently translated and either discarded (if it contains a stop codon) or kept as a valid in silico sequence. In the case of B-cell receptor simulations, SHM are introduced based on the user-defined parameters.

Supplementary Figure 2

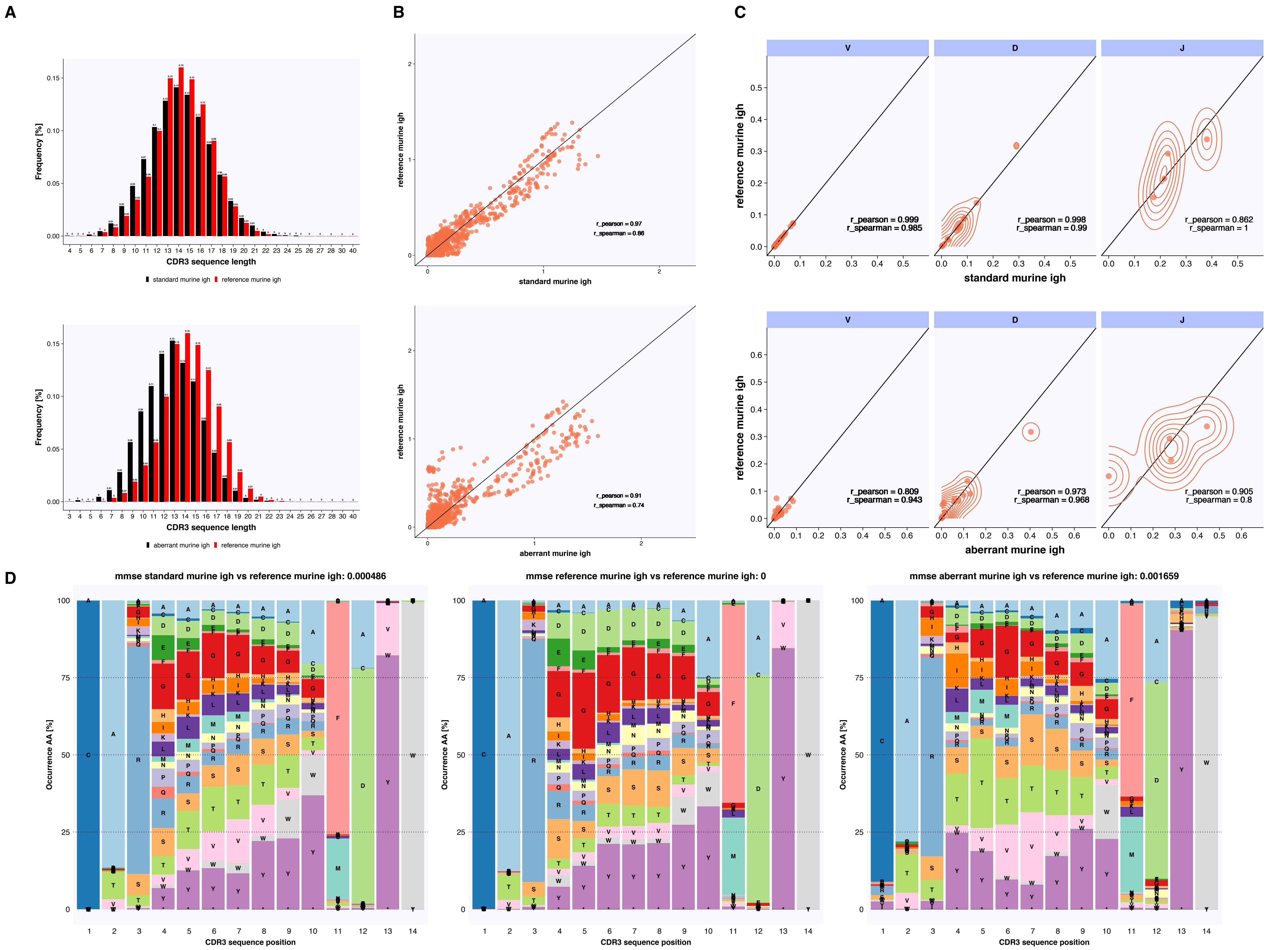

SFig. 2 immuneSIM simulates fully annotated murine IgH immune repertoires that are either high similar or deviating from experimental immune repertoires in regards to the major immune repertoire features length distribution, gapped-kmer occurrence, VDJ usage and CDR3 amino acid frequency.

(A) The CDR3 length distributions of a standard simulated murine IgH repertoire (simulated using default parameters) and experimental data (both annotated using IMGT) largely overlap, while the aberrant repertoire simulated with non-default parameters (Supplementary Table 2) shows a shift towards shorter lengths. (B) Gapped k-mer occurrence of CDR3 nucleotide sequence shows high correlation between default parameter simulation and experimental repertoires (upper panel, $r_{spearman} = 0.86$ for $k = 3$ and gap size $m \leq 3$, nkmers = 16384) and lower correlation to aberrant repertoires (lower panel, $r_{spearman} = 0.74$) . (C) The V, D and J frequencies between simulated (default parameters) and experimental repertoires are highly correlated ($r_{spearman} \geq 0.985$). Simulating aberrant repertoires is also possible (lower panel, $r_{spearman} \geq 0.8$). (D) The positional amino acid frequencies of CDR3 sequences (annotated using IMGT) of length 14 are shown to be highly similar (Mean of mean squared errors across positions, mmse: 0.000486) to experimental data (center). Repertoires simulated to be farther from the experimental dataset deviating with respect to positional amino acid frequency (mmse: 0.001659).

Supplementary Figure 3

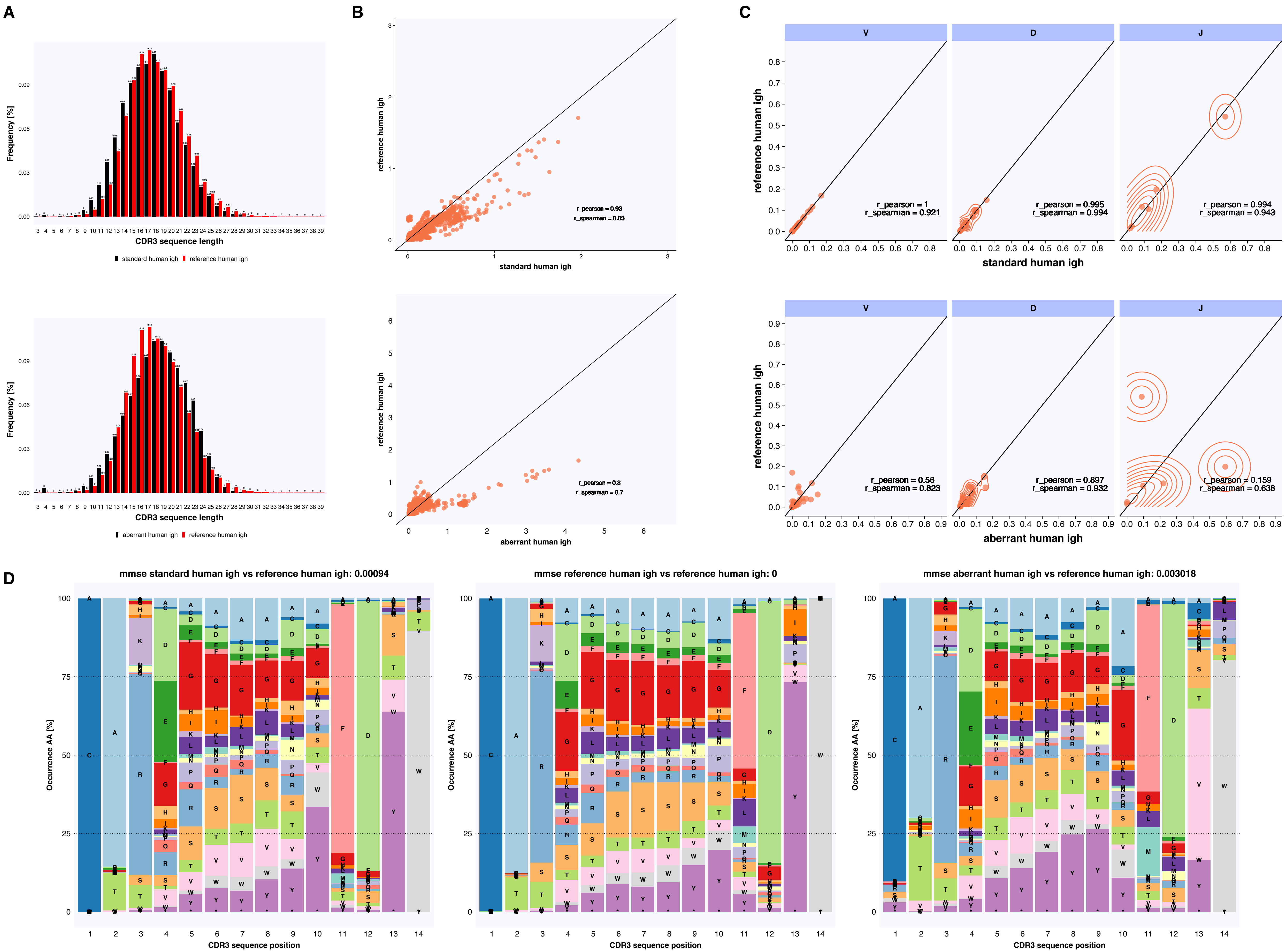

SFig. 3. Major features of standard and aberrant simulated human IgH repertoires. (A) The CDR3 length distributions of a standard simulated repertoires (simulated using default parameters) and experimental data (both annotated using IMGT) largely overlap while the aberrant repertoire simulated with non-default parameters (Supplementary Table 2) shows a shift towards shorter lengths. (B) Gapped k-mer occurrence of CDR3 nucleotide sequence shows high correlation between simulated (default parameters) and experimental repertoires (upper panel, $r_{spearman} = 0.83$ for $k = 3$ and gap size $m \leq 3$, nkmers = 16384) and lower correlation to aberrant repertoires (lower panel, $r_{spearman} = 0.7$) . (C) The V, D and J frequencies between simulated (default parameters) and experimental repertoires correlate to a high degree ($r_{spearman} \geq 0.921$). Simulating more deviating repertoires is also possible (lower panel, $r_{spearman} \geq 0.638$). (D) The positional amino acid frequencies of CDR3 sequences (annotated using IMGT) of length 14 are shown to be highly similar (Mean of mean squared errors across positions, mmse: 0.00094) to experimental data (center). Repertoires simulated to be farther from the experimental dataset deviating with respect to positional amino acid frequency (mmse: 0.003018).

Supplementary Figure 4

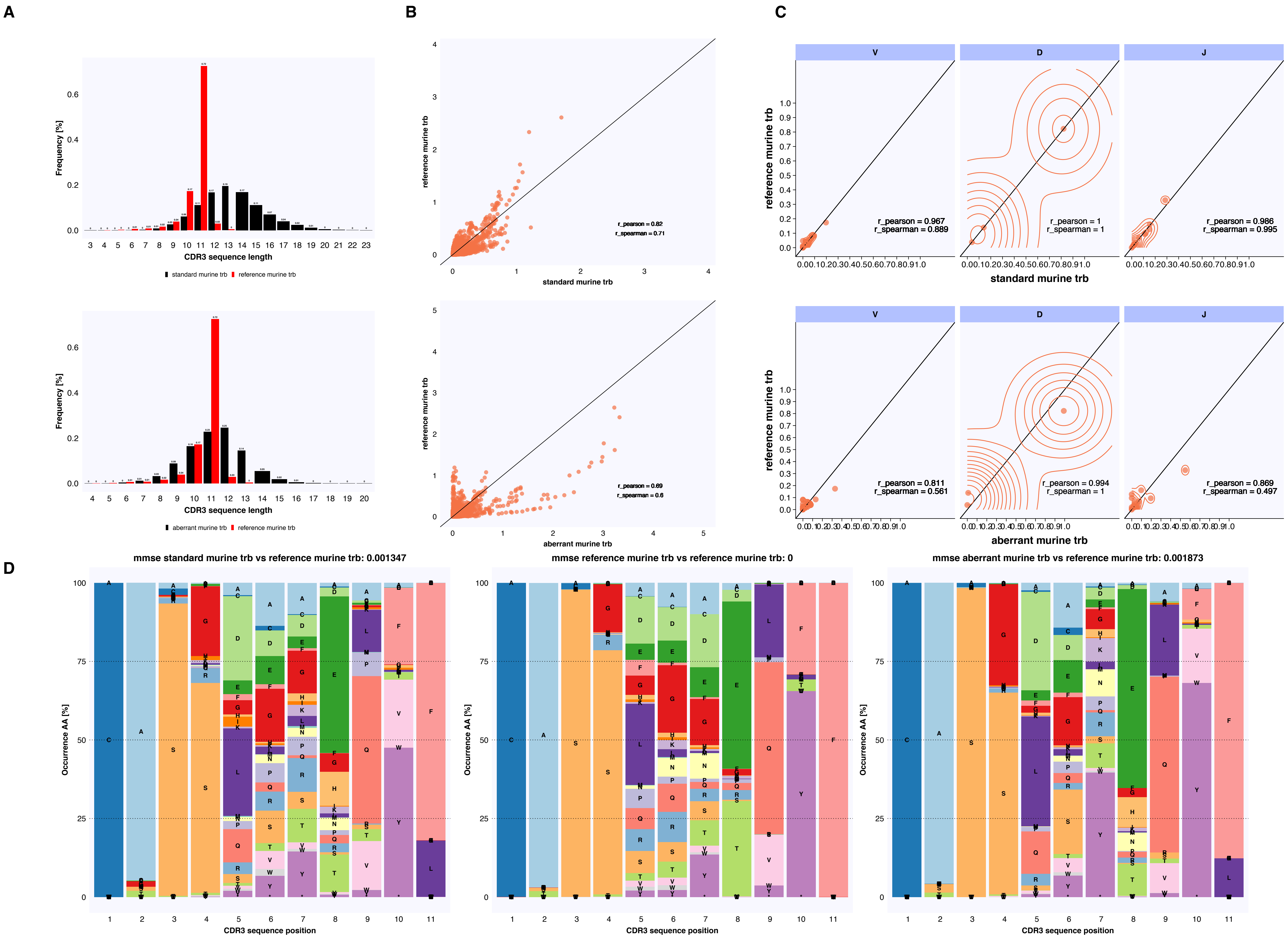

SFig. 4. Major features of standard and aberrant simulated murine TCRβ repertoires. The recovery of murine trb features are more difficult using immuneSIM due to the generally shorter sequence length. Nevertheless standard simulated repertoires (simulated using default parameters) and a aberrant repertoires simulated with non-default parameters (Supplementary Table 2) can be generated with different levels of similarity to experimental data. (A) The CDR3 length distributions of repertoires simulated with default parameters and experimental data (both annotated using IMGT) are shifted with the simulated repertoire having a more even distribution. (B) Gapped k-mer occurrence of CDR3 nucleotide sequence shows higher correlation between default parameter simulation and experimental repertoires (upper panel, $r_{spearman}$ = 0.71 for k = 3, m ≤ 3, nkmers = 16384) and low correlation to aberrant repertoires (lower panel, $r_{spearman}$ = 0.6) . (C) The V, D and J frequencies between simulated (default parameters) and experimental repertoires correlate to a high degree (upper panel, $r_{spearman}$ ≥ 0.889). Simulating more deviating repertoires is also possible (lower panel, $r_{spearman}$ ≥ 0.497). (D) The positional amino acid frequencies of CDR3 sequences (annotated using IMGT) of length 14 are shown to be highly similar (left, Mean of mean squared errors across positions, mmse: 0.001347) to experimental data (center). Repertoires simulated to be farther from the experimental dataset deviating with respect to positional amino acid frequency (right, mmse: 0.001873).

Supplementary Figure 5

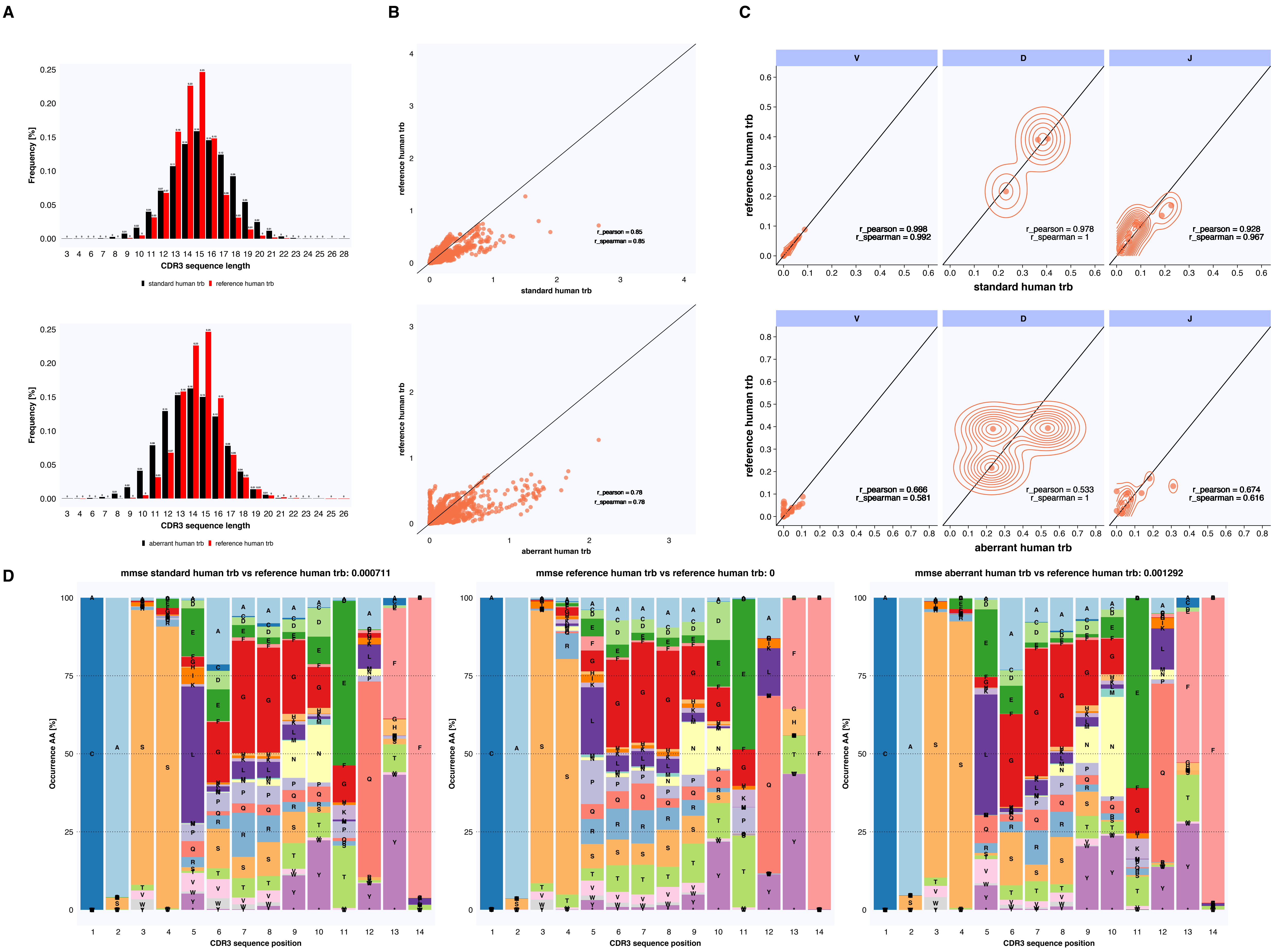

SFig. 5. Major features of standard and aberrant simulated human TCRβ repertoires. (A) The CDR3 length distributions of a standard repertoire (simulated using default parameters) and experimental data (both annotated using IMGT) largely overlap while the aberrant repertoire simulated with non-default parameters (Supplementary Table 2) shows a shift to shorter lengths. (B) Gapped k-mer occurrence of CDR3 nucleotide sequence shows high correlation between simulated (standard) and experimental repertoires (upper panel, $r_{spearman} = 0.85$ for $k = 3$, $m \leq 3$, nkmers = 16384) and lower correlation to aberrant repertoires (lower panel, $r_{spearman} = 0.78$) . (C) The V, D and J frequencies between standard simulated (default parameters) and experimental repertoires correlate to a high degree ($r_{spearman} \geq 0.967$). Simulating more deviating repertoires is also possible (lower panel, $r_{spearman} \geq 0.581$). (D) The positional amino acid frequencies of CDR3 sequences (annotated using IMGT) of length 14 are shown to be highly similar (Mean of mean squared errors across positions, mmse: 0.000711) to experimental data (center). Repertoires simulated to be farther from the experimental dataset deviating with respect to positional amino acid frequency (mmse: 0.001292).

Supplementary Figure 6

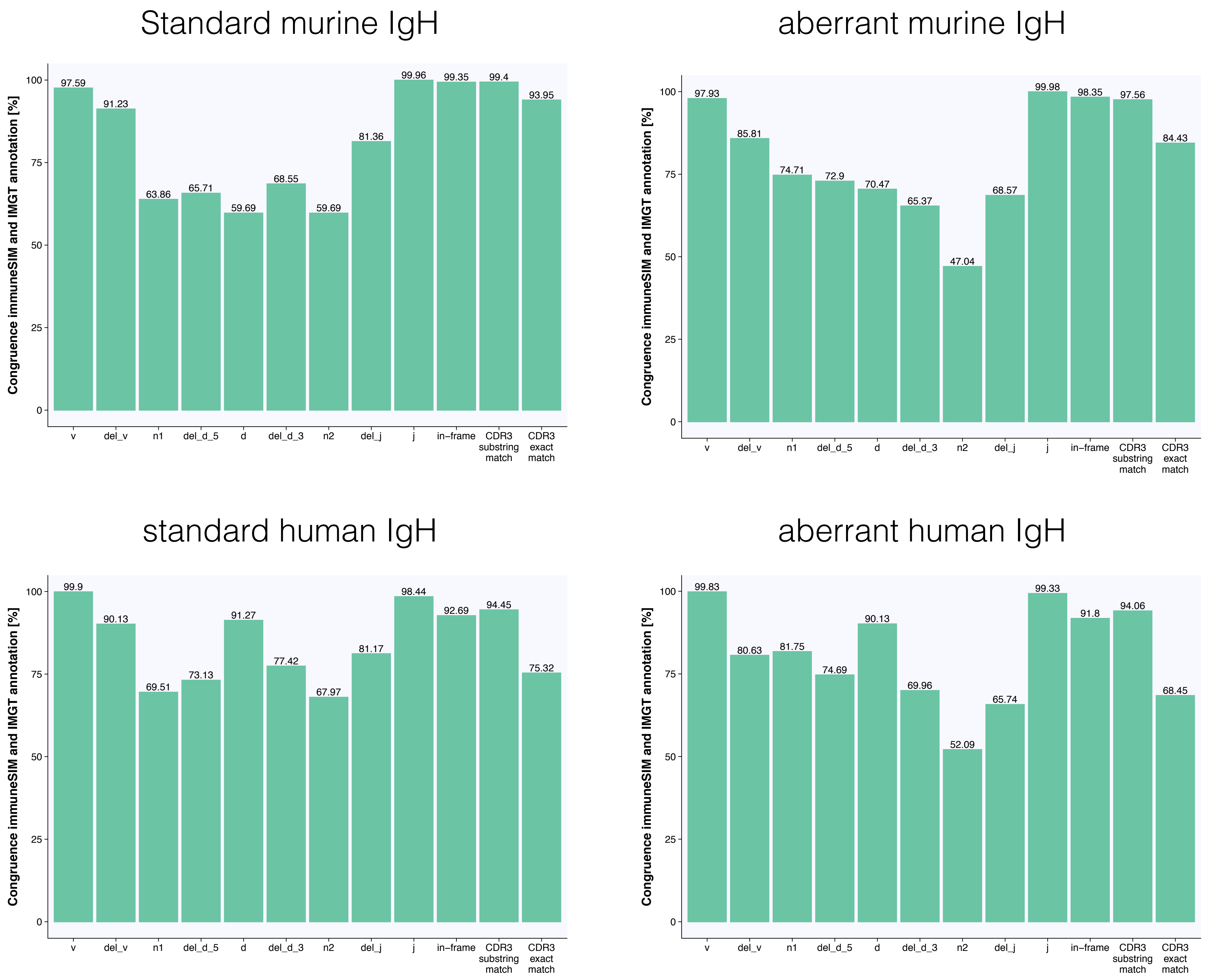

SFig. 6. ImmuneSIM generates B-cell receptor sequences that are in accord with IMGT annotation. Simulation of murine (top) and human (bottom) IgH repertoires each using default (standard, left) and non-default parameters (aberrant, right). The comparison of immuneSIM-annotated simulated repertoires with IMGT HighV-Quest annotation indicates that immuneSIM generates productive sequences with IMGT identifiable V,D,J genes, insertions, and deletions. The lower percent of congruence with respect to the n1-D-n2 portion of the CDR3 is expected due to the difficulty of D-gene annotation (Bolotin et al., 2015). Nearly all immuneSIM-simulated immune receptor sequences have an identifiable in-frame (>91.8%) CDR3 junction that is correctly annotated by immuneSIM to the degree that it is equal to or a substring of the IMGT CDR3 in >94.06% of all cases. N = 10’000.

Supplementary Figure 7

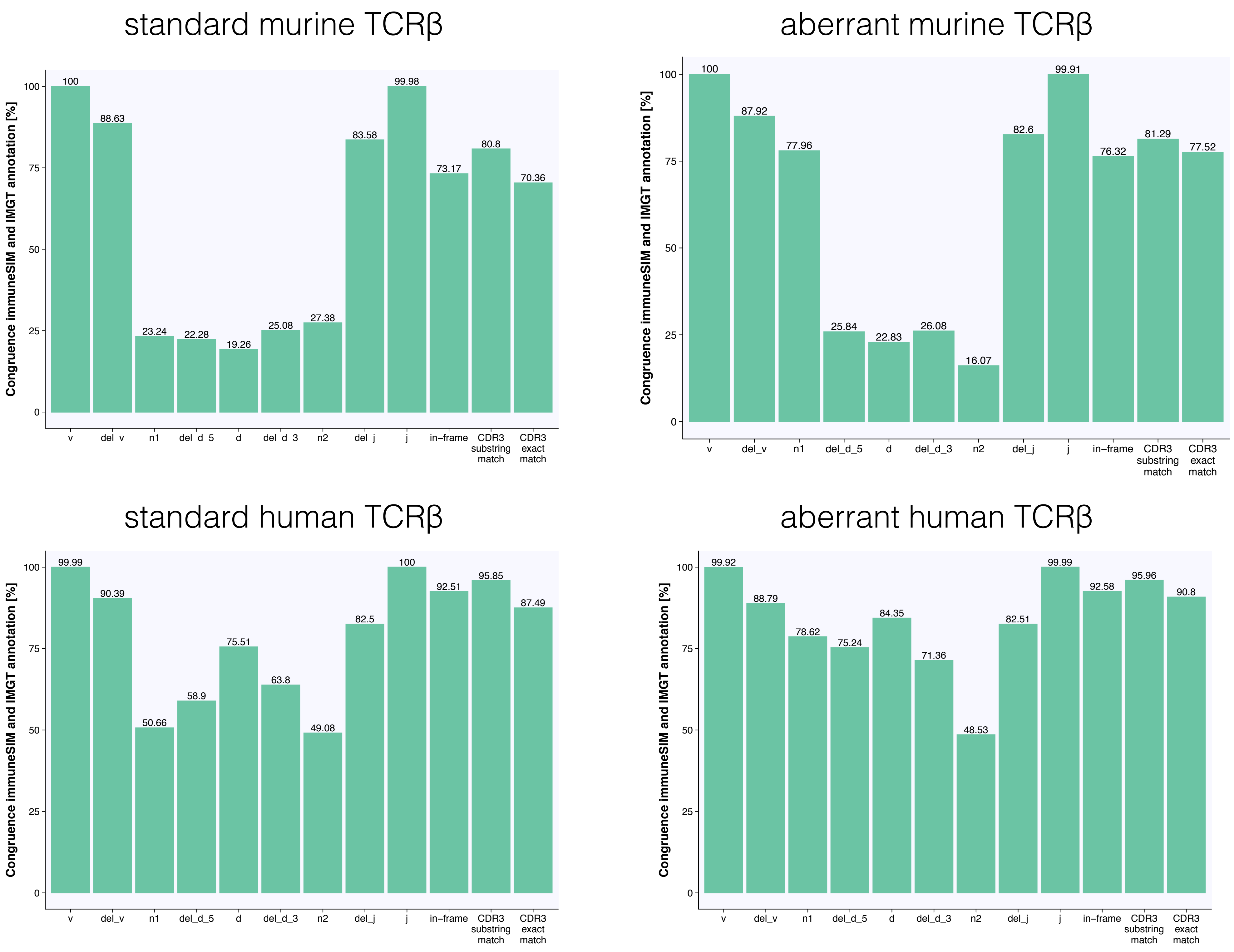

SFig. 7. High congruence with IMGT annotation is high for TCRβ repertoires across multiple species. Simulation of murine (top) and human (bottom) TCRβ repertoires each using default (standard, left) and non-default (aberrant, right) parameters. The comparison of immuneSIM-annotated simulated repertoires with IMGT HighV-Quest annotation indicates that immuneSIM generates productive sequences that can be annotated by IMGT. The performance is lowest for murine TCRβ, likely due to the increased difficulty the shorter CDR3s present especially with regard to D-gene annotation. However, immuneSIM repertoires have recognizable V and J genes (~100%) and >73% of sequences are determined to be in-frame with >70% exact CDR3 matches between IMGT and immuneSIM.

Supplementary Figure 8

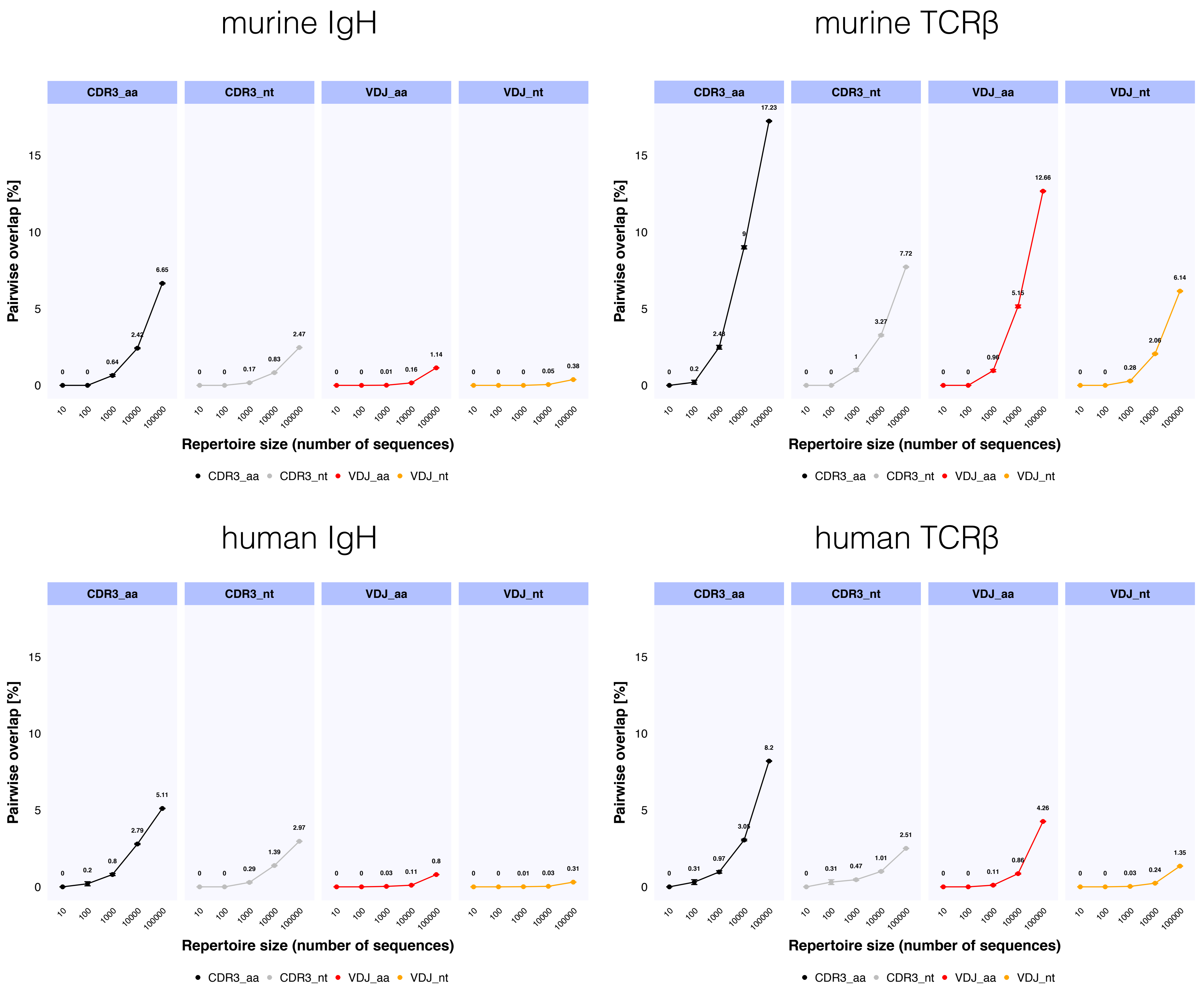

SFig. 8 The pairwise sequence overlap across simulated repertoires increases with repertoire size. Pairwise overlap between five repertoires per repertoire size and species/receptor chain combination was measured for amino acid and nucleotide CDR3 and VDJ sequences. Overlap reaches a maximum of 17.23% for the repertoire size of 100’000 murine TCRβ CDR3 amino acid sequences, confirming that immuneSIM produces highly diverse immune receptor repertoire sequences.

Supplementary Figure 9

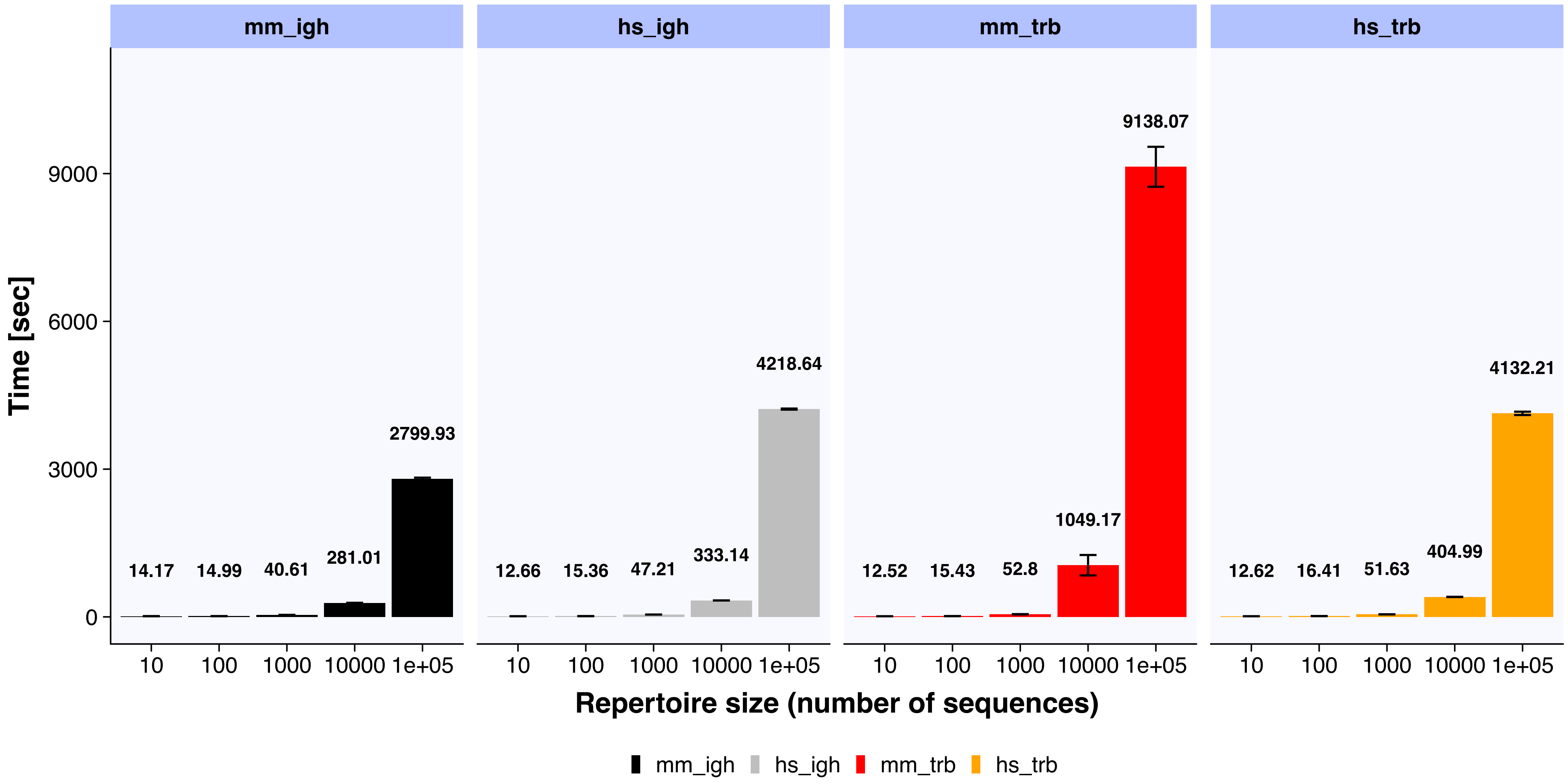

SFig.9 immuneSIM runtime differs for various species/receptor chain combinations. Murine and human IgH repertoires (mm_igh, hs_igh) can be simulated efficiently while in silico recombination of murine and human TCRβ repertoires (mm_trb, hs_trb) requires more time for larger datasets (due to lower likelihood of in-frame, productive recombination events). Runtime was measured as an average across five standard parameter simulations per size and category using the tictoc R package.
