# Supplementary material for: immuneSIM: tunable multi-feature simulation of B- and T-cell receptor repertoires for immunoinformatics benchmarking
Source: Bioinformatics. 2020 Apr 14;36(11):3594–6. doi: 10.1093/bioinformatics/btaa158 (PMC7334888; doi:10.1093/bioinformatics/btaa158)
Supplement: btaa158_Supplementary_Data [file btaa158_supplementary_data.zip › btaa158-Suppl_Data/04_immuneSIM_supplementary_data_rev2_unmarked.pdf]

Supplementary Data

Detailed Methods

A detailed explanation of each function of the immuneSIM R package is provided in the online documentation https://immuneSIM.readthedocs.io. Implementation details and procedures relevant to the understanding of the results (Supplementary Figures 2–9) presented in the Supplementary Data are described in more detail below.

Sampling of germline V, D, J genes

V, D and J genes are sampled based on V, D, J usage frequencies contained in the provided `list_germline_genes_allele_01` R list object contained in the package. The list contains IMGT germline gene sequences (Alamyar *et al.*, 2012) for human and murine immunoglobulin heavy and light and T-cell receptor beta and alpha chains as well as frequency distributions for each subset. In the case of immunoglobulin heavy and beta chain repertoires, the germline gene frequencies were obtained from published datasets (Greiff, Menzel, *et al.*, 2017; Madi *et al.*, 2017; DeWitt *et al.*, 2016; Emerson *et al.*, 2017), while uniform distributions were used in the case of light and beta chain repertoires due to a lack of fitting datasets (see Supplementary Table 1). The user is free to modify these frequencies or extend the `list_germline_genes_allele_01` to introduce different experimental frequencies or synthetic germline sequences. Since different V, D, J combinations have an unequal likelihood to form a productive in-frame sequence, a purely input-frequency-based sampling can lead to skewed V, D, J usage in simulated repertoires. Therefore, the `update_vdj_freqs` parameter allows for a re-adjustment of the initial VDJ frequencies after a defined threshold (default: 50% of the sequences) in order to generate sequences with a VDJ distribution that closely mirrors input frequencies.

Sampling of deletions and insertions

The deletion lengths are sampled from a provided R dataframe `insertions_and_deletion_lengths_df` which contains deletion lengths and insertion sequences pooled across all samples of a previously published mouse BCR study (Greiff, Menzel, *et al.*, 2017). The immuneSIM R package contains a subset of 500’000 entries, while the full dataset (11’363’603 entries) is available through GitHub (https://github.com/GreiffLab/immuneSIM) and can be loaded using the immuneSIM function `load_insdel_data()`. Each row in this reference dataset contains an experimentally observed insertion and deletion event. The provided data does not contain information on correlations between insertion and deletions and specific germline genes in order to enable a wider variety of possible recombinations of insertions, deletions, and germline genes. While immuneSIM does not provide a species or receptor chain specific reference dataset, the 11’363’603 data points for n1, n2 insertions, and V, D, J deletions cover a wide range of all theoretically possible insertion and deletion patterns. Analogously to the reference for the sampling of the V, D and J germline genes, the user has the option to modify or replace this reference file. The sampling of insertion and deletion starts with the sampling of the deletion lengths for the 3’ end of the V gene as well as on 5’ and 3’ end of the D gene from `insertions_and_deletion_lengths_df`. The deletion at the 5’ J region is applied after a valid anchor point is identified – J-TRP and J-PHE (represented by `"tgg"` and `"ttt", "ttc"`, respectively) (Lefranc, 2011). Specifically, the deletion is applied to the pre-anchor subsequence of the chosen J gene. For each deletion length, an additional checkpoint ensures that they are not longer than the sequence they are applied to. After deletion lengths have been sampled and applied, the insertions n1 and n2 are sampled from a subset of `insertions_and_deletion_lengths_df` which is restricted to insertions of lengths that will result in an in-frame CDR3 (complementarity determining region 3). This represents a simplified model of insertions and deletions as there is (as mentioned above) no linkage between insertion and deletion events and germline-genes. Thus, the model does not reflect germline gene differences in end trimming as was observed previously in experimental data (Thörnqvist and Ohlin, 2018). Further, the model does not distinguish between insertion and deletion events that occur during VDJ recombination and those that can occur during somatic hypermutation. Finally, we would like to note that the method described here is intentionally restrictive in regards to non-productive sequences focusing on the simulation of repertoires containing productive, in-frame sequences.

Identifying the CDR3 junctional region

The junctional region of the immune receptor sequence, at which the V, D and J genes are brought together, forms the CDR3. During the simulation of each sequence, the CDR3 is determined based on the V-gene cysteine anchor (represented by `"tgt", "tgc"`) and the J-gene anchors J-TRP, J-PHE (Lefranc, 2011). This CDR3 is translated into amino acids and matched to the full sequence. If the sequence is productive (in-frame, no stop codons) an additional check is performed to determine whether the determined J-gene anchor is correct by looking for additional occurrences of out-of-frame patterns (as identified via High-VQuest analysis of simulated sequences) further downstream of the sequence. This check for out-of-frame patterns ensures that cases in which there are additional W (such as in IGHJ2 and IGHJ5) or F (in the case of IGKJ3 and IGLJ4) do not lead to assignment problems. Following these checks, a further check ensures that the amino acid length of the CDR3 detected is not above or below a user-set threshold.

Motif implantation

Since sequence motifs have been previously shown to be predictive of public and private clones (Greiff, Weber, *et al.*, 2017) as well as antigen-binding and disease course (Dash *et al.*, 2017; Glanville *et al.*, 2017; Ostmeyer *et al.*, 2019; Akbar *et al.*, 2019), controlled and recoverable implantation of motifs is essential for the benchmarking of machine learning methods. immuneSIM enables the user to implant user-defined or randomly generated k-mer motifs at various frequencies in the nucleotide and amino acid CDR3 sequences. Additionally, there is an option to choose between positional and

position-agnostic implantation. The immuneSIM function `motif_implantation` takes the user input consisting of an AIRR-compliant repertoire (Rubelt *et al.*, 2017), a list defining the motifs, their frequencies and a position parameter and implants the motif in the CDR3 of the nucleotide and amino acid sequences of the repertoire. At the implantation position, the existing nucleotides and amino acids are replaced, thus conserving the length of the modified CDR3.

Synonymous codon replacement

In order to create repertoires that have 100% amino acid sequence identity but differ in their nucleotide composition (relevant for testing the predictive power of nucleotide and amino acid sequence-based methods), immuneSIM provides the function `codon_replacement`, which allows for the substitution of nucleotide codons. This can be relevant for testing predictive performance of methods for which both amino acid-based features (such as amino acid frequency distributions) as well as nucleotide-based features (e.g., gapped-k-mer occurrence) can be used as input. Specifically, an example of such a use case would be the prediction of public and private clones using Support Vector Machines (Greiff, Weber, *et al.*, 2017). The function takes as input (i) an AIRR-compliant repertoire (Rubelt *et al.*, 2017), (ii) the sequences that should be modified ("AA", "nt" or "both"), (iii) a list containing the user-defined rules for replacement (i.e. the codons that should be replaced and their replacements) and (iv) a probability with which a sequence in the repertoire should be skipped in the replacement process. Based on the probability defined in (iv), each sequence is either marked as a target sequence (codon replacement is applied) or kept as is (no codon replacement occurs). The to-be-replaced codons are identified in each target sequence and all of them are replaced according to the rules specified in the user-provided list. While the main intended use case for the `codon_replacement` function is for synonymous replacement, it supports any kind of nucleotide replacement and can, therefore, also be used for nonsynonymous codon replacements that leads to a modification of the amino acid sequence.

Architecture modification

The repertoire architecture as defined by the properties of the similarity network of its amino acid CDR3 sequences has been shown to be a reproducible and robust feature across B- and T- cell repertoires (Madi *et al.*, 2017; Miho *et al.*, 2019). The immuneSIM function `hub_seqs_exclusion` enables the modification of the similarity architecture through the deletion of the top X (percentage, defined by user) hub sequences (i.e. sequences of connective importance in the network). This is achieved by calculating and analyzing the CDR3 similarity network based on the Levenshtein distance (edit distance = 1) using the R packages igraph (Csardi and Nepusz, 2006) and stringdist (Loo, 2014). The top hub sequences are identified based on their hub score and excluded.

Simulation of somatic hypermutation

The simulation of somatic hypermutation (SHM) is performed using the previously published AbSim R package (Yermanos *et al.*, 2017). After the sampling of the V, D, J genes and insertions and deletions, SHM is performed based on the user-defined mode and SHM probability (for details see package documentation). Each SHM event is recorded in a character string that is output in the repertoire dataframe. immuneSIM additionally provides a function `shm_event_reconstruction` which decodes the SHM strings into a list of dataframes with the column names "location", "pre_SHM_nt" and "post_SHM_nt". The integration of our previously published method provides an in-package method to create somatically hypermutated repertoires. That said, if the user wishes to model aspects of SHM simulation not taken into account by AbSIM (Sheng *et al.*, 2017; Kirik *et al.*, 2017; Schramm and Douek, 2018; Guo *et al.*, 2019), any immuneSIM repertoire can be used as input for any other SHM simulation methods (Hoehn *et al.*, 2019) that take AIRR standard repertoires as input.

immuneSIM random

The random mode allows for the simulation of fully random nucleotide and amino acid sequences of a specified length distribution. These sequences do not take into account V, D, J germline gene sequences and insertion or deletion information and are thus VDJ germline gene agnostic and can serve as a simple negative control. The output repertoire dataframe thus only contains information in the sequence and clone count columns of the repertoire dataframe while the remaining columns are set to `NA`.

Modification of V,(D),J usage

immuneSIM provides three ways of introducing modified V, D, J frequencies into repertoires. First, through the modification of the reference list `list_germline_genes_allele_01` used for germline gene sampling. A second option is provided by the `vdj_dropout` parameter of the immuneSIM function lets the user drop a chosen number of V, D and J genes (i.e., by setting their frequencies to zero). Finally, the user can also introduce noise into the VDJ frequencies via the parameter `vdj_noise` in the immuneSIM function. The noise value may be chosen between 0 (no noise) and 1 (recommended maximum noise) and sets the standard deviation for a normal distribution (mean = 0, bounded by -1,1) from which noise terms are sampled. Each sampled noise term is scaled by the frequency of the current germline gene and added to it (multiplicative noise). The `vdj_noise`, therefore, leads to a smaller modification of the germline gene frequencies than the first two options.

Simulation of clonal abundance

Clonal abundance, shown to be a static representation of the clonal dynamics of immune repertoires that follows a power-law distribution (Greiff *et al.*, 2015; Greiff, Menzel, *et al.*, 2017), is simulated using the poweRlaw R package (Gillespie, 2015). Subsequently, the distribution of frequencies is transformed into counts by setting the lowest frequency to count = 1 (singletons). Unlike the simulated sequences, these counts are not based on

experimental data. Therefore, there is no connection between a given sequence and its count as it is the case for the synthetic data provided by IGoR (Marcou *et al.*, 2018). The user can set the alpha parameter that controls the evenness of the power-law distribution and also has the choice to create a clone abundance that is equal across all clones (uniform distribution).

Paired repertoires

The simulation of paired repertoires (heavy and light chain B-cell receptor repertoires, beta and alpha chain T-cell receptor repertoires) is achieved by simulating repertoires for heavy and light (or beta and alpha) receptor chains separately and combining them post-simulation using the immuneSIM function `combine_into_paired` which combines the repertoires and renames the columns. As the clonal frequencies are simulated in a sequence agnostic manner the heavy chain frequencies are kept while the frequencies from the light chain simulation discarded. The pairing occurs randomly as there is as of today only limited reliable information available on the preferential associations of chain pairing (Shcherbinin *et al.*, 2019; Zhou and Kleinstein, 2019).

Simulation of standard and aberrant repertoires

The repertoires described in Supplementary Figures 2–5 contain 10'000 simulated sequences each. For the standard repertoires, default parameters of immuneSIM (representing experimental germline gene frequencies, no somatic hypermutation, no restrictions on insertion and deletions) were used. For the aberrant repertoires V, D, J usage was modified by introducing noise (`vdj_noise` = 0.2) and `vdj_dropout` depending on available germline genes (mm_igh: V = 10, D = 3, J = 1, hs_igh: V = 10, D = 4, J = 2, mm_trb: V= 10, D = 1, J = 5 and hs_trb: V = 10, D = 0, J = 2). Additionally, insertions and deletions were restricted by disallowing n1 insertions (`ins_del_dropout = "no_insertions_n1"`). Finally, for IgH repertoires SHM was introduced (`shm.mode = "data", shm.prob =15/350`) (See also Supplementary Table 2).

Determining congruence with IMGT annotation

The full nucleotide VDJ sequences of each simulated repertoire were uploaded to and annotated with IMGT High-VQuest (Aouinti *et al.*, 2015). Following annotation, the percentage of exactly recovered immuneSIM V, D and J calls and the insertions and deletions by High-VQuest were determined. Further, the percentage of simulated sequences considered as in-frame and productive by IMGT as well as the substring and full string overlap of CDR3 sequences was calculated.

Statistical analysis and plots

Statistical analysis was performed using R 3.4.0 (R Core Team). Graphics were generated using the ggplot2 (Wickham, 2009), ggthemes (Arnold, 2019), ComplexHeatmap (Gu *et al.*, 2016), circlize (Gu *et al.*, 2014) and RColorBrewer (Neuwirth, 2014) R packages and the ggplot2 theme theme.akbar (Akbar, 2019). The Supplementary Figures 2–5 were generated using the immuneSIM functions `plot_repertoire_A_vs_B` and `plot_report_repertoire`.

Positional amino acid distribution

The amino acid frequencies were determined per position across all sequences of each CDR3 length. For the comparison of amino acid frequency distributions, the mean of the mean squared error (mmse) between distributions was calculated per position as previously described (Mason *et al.*, 2018). For the comparison between simulated and experimental repertoires, the CDR3s as annotated by IMGT High-VQuest (Aouinti *et al.*, 2015) were used.

Gapped k-mer occurrence

For each repertoire, the occurrence of gapped k-mers was calculated across all CDR3 nucleotide sequences for parameters ($k = 3$, $m \leq 3$, where k is the k-mer amino acid length and m is the number of amino acid gaps), as described by Palme *et al.*, 2015. The gapped-kmers are counted for the core of the CDR3 only, excluding the first three and last two amino acids containing more conserved patterns. The gapped k-mer occurrences were compared between simulated and experimental reference datasets (see Supplementary Table 1) and the Pearson and Spearman correlation was calculated. For the comparison between simulated and experimental repertoires, the CDR3s as annotated by IMGT High-VQuest (Aouinti *et al.*, 2015) were used.

V, D, J germline gene usage

The usage of IMGT germline genes was compared between simulated and experimental reference datasets (see Supplementary Tables 1 and 2) and the Pearson and Spearman correlation coefficient was calculated. For this comparison, the V, D, J germline gene annotation from the immuneSIM output was used.

Runtime and repertoire overlap

In order to evaluate the runtime performance of immuneSIM, five repertoires were generated using default parameters for each category (murine igh, murine trb, human igh, human trb) and repertoire size (10, 100, 1'000, 10'000, 100'000 sequences). The runtime for the generation of each repertoire was measured using the tictoc R package (Izrailev, 2019) in R version 3.5 (R Core Team) on an iMac (Late 2013, Processor: 2.9 GHz Intel Core i5, Memory: 16GB 1600 MHz DDR3, macOS Sierra v 10.12.6). Subsequently, the repertoire overlap was calculated for each category across the five simulated repertoires as previously described (Greiff, Weber, *et al.*, 2017).

Features and specifications of the immuneSIM simulation process

Features: immuneSIM provides a user-friendly end-to-end traceable method to create synthetic B- and T-cell immune receptor repertoires in R by calling a single function that allows for multi-parameter tuning across a wide range of properties and feature distributions. All features of immuneSIM are detailed in-depth in the main text and Fig 1 of the Application Note. In addition, Supplementary Table 3 provides an overview of the features of immuneSIM in comparison to other immune receptor repertoire simulation software suites (Marcou *et al.*, 2018; Sethna *et al.*, 2019; Safonova *et al.*, 2015; Hoehn *et al.*, 2019). To keep simulation speed and useability high, we opted for some simplifications in the simulation process. The most notable simplifications are: (i) The insertion and deletion events occurring in the immuneSIM simulations are based on the insertions and deletions identified in high-throughput sequencing datasets. As such the simulation of insertions and deletions in immuneSIM combines two types of insertion/deletion events, namely those that occur during VDJ recombination and those that can occur as a consequence of SHM. Specifically, the end trimming is not simulated in a germline-gene specific manner as this would lead to an extraordinarily specific and restrictive simulation. Rather, we conduct the deletion randomly and rely on the in-frame and productivity tests performed downstream to correct for any non-appropriate trimmings (see SFig1). (ii) The identification of J-gene anchors as described in Supplementary Fig. 1 necessitates additional checks (see "Identifying the CDR3 region") due to the occurrence of additional W (in IGHJ2 and IGHJ5) and F (as in IGKJ3 and IGLJ4). (iii) There is no single parameter in the current version that would allow creating a repertoire composition with varying degrees of mutations (ie an 'in-between set') in a single simulation run. It can, however, be achieved by an iterative approach of simulating repertoires based on different parameter sets and subsequently combining them into a mixed 'in-between set' post-simulation. (iv) The sequence counts are simulated separately from the sequences and randomly assigned. (v) immuneSIM does not simulate FASTQ/FASTA files but fully processed annotated repertoires and therefore does not output non-productive sequences. (vi) immuneSIM does not exclude all potentially unrealistic sequences (ie it does not provide a blacklist of prohibited VDJ pairings). (vii) The simulation of SHM is based on the previously published AbSIM method (Yermanos et al., 2017) beyond which immuneSIM does not claim to provide novel insight or methods related to SHM simulation.

Reference data

Germline gene sequences for T-cell receptor beta and alpha chains and B-cell receptor heavy and light chains were obtained from the IMGT reference directory (Alamyar et al., 2012) on July 31, 2019, and collected in a reference R list. Subsequently, V, D, J usage frequencies from published data were added for human and murine T-cell receptor beta and B-cell receptor heavy chain germline genes while uniform distributions were added to human and murine T-cell receptor alpha and B-cell receptor light chain germline genes (see Supplementary Table 1, below). The use of different germline gene reference data by the user is possible through the modification of the reference list `list_germline_genes_allele_01`. Simulating repertoires using different reference data based on experimental data based on various isotypes or sequencing technology could also be used to simulate repertoires that are biased to reflecting isotype-specific characteristics or sequencing technology-specific artifacts.

**Supplementary Table 1. Published datasets for germline gene frequencies used in immuneSIM.**				
**Species**	**Receptor chain**	**Sample**	**Dataset**	**Number of sequences**
mus musculus	IgH	healthy_2_nfbc_igm	Greiff *et al.*, 2017	373’993
mus musculus	Igκ/λ	-	uniform distribution	NA
mus musculus	TCRβ	SRR1339480_Untreated	Madi *et al.*, 2017	17’752
mus musculus	TCRα	-	uniform distribution	NA
homo sapiens	IgH	D1_Na	DeWitt *et al.*, 2016	2’557’564
homo sapiens	Igκ/λ	-	uniform distribution	NA
homo sapiens	TCRβ	HIP19048	Emerson *et al.*, 2017	52’200
homo sapiens	TCRα	-	uniform distribution	NA

Supplementary Table. 1. ImmuneSIM provides germline gene frequencies based on four published datasets. For the simulation of T-cell receptor alpha chains and B-cell receptor light chains, immuneSIM utilizes uniform sampling of IMGT germline genes since the number of available datasets is currently too limited to compute reliable germline frequencies.

**Supplementary Table 2. Parameters for standard and aberrant repertoires. (SFig. 2—7)**						
**Name**	**species, receptor, chain**	**vdj_noise**	**vdj_dropout**	**ins_del_dropout**	**shm.mode**	**shm.prob**
standard murine igh	“mm”, “ig”, “h”	0	V = 0, D = 0, J = 0	“”	“none”	-
aberrant murine igh	“mm”, “ig”, “h”	0.2	V = 10, D = 3, J = 1	“no_insertions_n1”	“data”	15/350
standard murine trb	“mm”, “tr”, “b”	0	V = 0, D = 0, J = 0	“”	“none”	-
aberrant murine trb	“mm”, “tr”, “b”	0.2	V = 10, D = 1, J = 5	“no_insertions_n1”	“none”	-
standard human igh	“hs”, “ig”, “h”	0	V = 0, D = 0, J = 0	“”	“none”	-
aberrant human igh	“hs”, “ig”, “h”	0.2	V =10, D = 4, J = 2	“no_insertions_n1”	“data”	15/350
standard human trb	“hs”, “tr”, “b”	0	V = 0, D = 0, J = 0	“”	“none”	-
aberrant human trb	“hs”, “tr”, “b”	0.2	V = 10, D = 0, J = 2	“no_insertions_n1”	“none”	-

Supplementary Table. 2. Parameters for the simulation of standard and aberrant repertoires. Standard repertoires were simulated using default parameters based on the experimental reference datasets. For the aberrant repertoires, noise was introduced into the VDJ germline frequencies, VDJ germline genes were dropped, n1 insertions were disallowed and for aberrant IgH repertoires SHM was introduced.

Supplementary Table 3. Comparison of immune repertoire simulation software suites.

	**ImmuneSIM**	**IgOR/OLGA**	**IgSimulator**	**Immcantation*** (*not primarily a simulation tool)
Publication	This publication.	(Marcou *et al.*, 2018; Sethna *et al.*, 2019)	(Safonova *et al.*, 2015)	(Hoehn *et al.*, 2019; Gupta *et al.*, 2015; Heiden *et al.*, 2014; Nouri and Kleinstein, 2018; Gadala-Maria *et al.*, 2015)
Platform	R	C++, Python	C++,Python	R,Python
Input	Preset simulation parameters for species/chain combinations or input of user-defined parameters for germline genes, insertions/deletions, noise terms, diversity distribution and post recombination repertoire modifications.	Preset simulation parameters for species/chain combinations or immune receptor sequences for recombination model inference.	Simulation parameters include repertoire size, chain type, expected number of mutated sequences.	FASTQ (but depends on the specific analysis step within the Immcantation suite)
Output format of synthetic sequences	• Synthetic immune receptor (BCR/TCR) sequences with per-sequence simulation events by default<br>• AIRR-compliant	• Synthetic immune receptor (BCR/TCR)) sequences<br>• Not AIRR compliant	• FASTA/Q-files of synthetic BCR sequences<br>• Not AIRR-compliant	AIRR-compliant output
Simulation of (native-like) repertoires	Yes	Yes	Yes	No
Modification of germline gene frequencies	• Insertion of experimental data<br>• Addition of noise to frequencies<br>• Dropout of germline genes	Not available as option via the command line	No	No
Insertion and deletion	Based on observed insertion and deletion events	Based on observed insertion and deletion events	Random insertions and deletions	No
Implantation of sequence motifs	Yes	No	No	No
Generation of repertoires with modified sequence similarity architecture	Yes	No	No	No
Simulation of SHM	Yes, based on AbSIM (Yermanos *et al.*, 2017)	No	Yes	Yes
Integrated (basic or advanced) analysis of simulated repertoires	Yes	No	Yes (Visualization of simulated repertoire stats)1	Yes

*** ImmuneSIM (green), IgOR/OLGA (yellow), IgSimulator (pink), and Immcantation (blue).**

References

Akbar,R. *et al.* (2019) A finite vocabulary of antibody-antigen interaction enables predictability of paratope-epitope binding. *bioRxiv*, 759498.

Akbar,R. (2019) themeakbar Zenodo.

Alamyar,E. *et al.* (2012) IMGT® Tools for the Nucleotide Analysis of Immunoglobulin (IG) and T Cell Receptor (TR) V-(D)-J Repertoires, Polymorphisms, and IG Mutations: IMGT/V-QUEST and IMGT/HighV-QUEST for NGS. In, Christiansen,F.T. and Tait,B.D. (eds), *Immunogenetics: Methods and Applications in Clinical Practice*, Methods in Molecular Biology. Humana Press, Totowa, NJ, pp. 569–604.

Aouinti,S. *et al.* (2015) IMGT/HighV-QUEST Statistical Significance of IMGT Clonotype (AA) Diversity per Gene for Standardized Comparisons of Next Generation Sequencing Immunoprofiles of Immunoglobulins and T Cell Receptors. *PLoS ONE*, **10**, e0142353.

Arnold,J.B. (2019) ggthemes: Extra Themes, Scales and Geoms for 'ggplot2'.

Csardi,G. and Nepusz,T. (2006) The igraph software package for complex network research. *InterJournal*, **Complex Systems**, 1695.

Dash,P. *et al.* (2017) Quantifiable predictive features define epitope-specific T cell receptor repertoires. *Nature*, **547**, 89–93.

DeWitt,W.S. *et al.* (2016) A Public Database of Memory and Naive B-Cell Receptor Sequences. *PLOS ONE*, **11**, e0160853.

Emerson,R.O. *et al.* (2017) Immunosequencing identifies signatures of cytomegalovirus exposure history and HLA-mediated effects on the T cell repertoire. *Nat. Genet.*, **49**, 659–665.

Gadala-Maria,D. *et al.* (2015) Automated analysis of high-throughput B-cell sequencing data reveals a high frequency of novel immunoglobulin V gene segment alleles. *Proc. Natl. Acad. Sci.*, **112**, E862–E870.

Gillespie,C.S. (2015) Fitting Heavy Tailed Distributions: The poweRlaw Package. *J. Stat. Softw.*, **64**, 1–16.

Glanville,J. *et al.* (2017) Identifying specificity groups in the T cell receptor repertoire. *Nature*, **547**, 94–98.

Greiff,V. *et al.* (2015) A bioinformatic framework for immune repertoire diversity profiling enables detection of immunological status. *Genome Med.*, **7**.

Greiff,V., Weber,C.R., *et al.* (2017) Learning the High-Dimensional Immunogenomic Features That Predict Public and Private Antibody Repertoires. *J. Immunol. Baltim. Md 1950*, **199**, 2985–2997.

Greiff,V., Menzel,U., *et al.* (2017) Systems Analysis Reveals High Genetic and Antigen-Driven Predetermination of Antibody Repertoires throughout B Cell Development. *Cell Rep.*, **19**, 1467–1478.

Gu,Z. *et al.* (2014) circlize Implements and enhances circular visualization in R. *Bioinforma. Oxf. Engl.*, **30**, 2811–2812.

Gu,Z. *et al.* (2016) Complex heatmaps reveal patterns and correlations in multidimensional genomic data. *Bioinforma. Oxf. Engl.*, **32**, 2847–2849.

Guo,Y. *et al.* (2019) cAb-Rep: A Database of Curated Antibody Repertoires for Exploring antibody diversity and Predicting Antibody Prevalence. *bioRxiv*, 765099.

Gupta,N.T. *et al.* (2015) Change-O: a toolkit for analyzing large-scale B cell immunoglobulin repertoire sequencing data. *Bioinformatics*, btv359.

Heiden,J.A.V. *et al.* (2014) pRESTO: a toolkit for processing high-throughput sequencing raw reads of lymphocyte receptor repertoires. *Bioinformatics*, **30**, 1930–1932.

Hoehn,K.B. *et al.* (2019) Repertoire-wide phylogenetic models of B cell molecular evolution reveal evolutionary signatures of aging and vaccination. *Proc. Natl. Acad. Sci.*, **116**, 22664–22672.

Kirik,U. *et al.* (2017) Antibody Heavy Chain Variable Domains of Different Germline Gene Origins Diversify through Different Paths. *Front. Immunol.*, **8**.

Lefranc,M.-P. (2011) From IMGT-ONTOLOGY DESCRIPTION Axiom to IMGT Standardized Labels: For Immunoglobulin (IG) and T Cell Receptor (TR) Sequences and Structures. *Cold Spring Harb. Protoc.*, **2011**, pdb.ip83.

Loo,M.P.J. van der (2014) The stringdist Package for Approximate String Matching. *R J.*, **6**, 111–122.

Madi,A. *et al.* (2017) T cell receptor repertoires of mice and humans are clustered in similarity networks around conserved public CDR3 sequences. *eLife*, **6**, e22057.

Marcou,Q. *et al.* (2018) High-throughput immune repertoire analysis with IGoR. *Nat. Commun.*, **9**, 561.

Mason,D.M. *et al.* (2018) High-throughput antibody engineering in mammalian cells by CRISPR/Cas9-mediated homology-directed mutagenesis. *Nucleic Acids Res.*

Miho,E. *et al.* (2019) Large-scale network analysis reveals the sequence space architecture of antibody repertoires. *Nat. Commun.*, **10**, 1321.

Neuwirth,E. (2014) RColorBrewer: ColorBrewer Palettes.

Nouri,N. and Kleinstein,S.H. (2018) A spectral clustering-based method for identifying clones from high-throughput B cell repertoire sequencing data. *Bioinformatics*, **34**, i341–i349.

Ostmeyer,J. *et al.* (2019) Biophysicochemical Motifs in T-cell Receptor Sequences Distinguish Repertoires from Tumor-Infiltrating Lymphocyte and Adjacent Healthy Tissue. *Cancer Res.*, **79**, 1671–1680.

Palme,J. *et al.* (2015) KeBABS: an R package for kernel-based analysis of biological sequences: Fig. 1. *Bioinformatics*, **31**, 2574–2576.

R Core Team R: A Language and Environment for Statistical Computing R Foundation for Statistical Computing, Vienna, Austria.

Rubelt,F. *et al.* (2017) Adaptive Immune Receptor Repertoire Community recommendations for sharing immune-repertoire sequencing data. *Nat. Immunol.*

Safonova,Y. *et al.* (2015) IgSimulator: a versatile immunosequencing simulator. *Bioinformatics*, btv326.

Schramm,C.A. and Douek,D.C. (2018) Beyond Hot Spots: Biases in Antibody Somatic Hypermutation and Implications for Vaccine Design. *Front. Immunol.*, **9**.

Sethna,Z. *et al.* (2019) OLGA: fast computation of generation probabilities of B- and T-cell receptor amino acid sequences and

motifs. *Bioinformatics*, **35**, 2974–2981.
Shcherbinin,D.S. *et al.* (2019) Comprehensive analysis of structural and sequencing data reveals almost unconstrained chain pairing in TCRαβ complex. *bioRxiv*, 693630.
Sheng,Z. *et al.* (2017) Gene-Specific Substitution Profiles Describe the Types and Frequencies of Amino Acid Changes during Antibody Somatic Hypermutation. *Front. Immunol.*, **8**.
Thörnqvist,L. and Ohlin,M. (2018) The functional 3'-end of immunoglobulin heavy chain variable (IGHV) genes. *Mol. Immunol.*, **96**, 61–68.
Wickham,H. (2009) ggplot2: Elegant Graphics for Data Analysis Springer-Verlag New York.
Yermanos,A. *et al.* (2017) Comparison of methods for phylogenetic B-cell lineage inference using time-resolved antibody repertoire simulations (AbSim). *Bioinformatics*.
Zhou,J.Q. and Kleinstein,S.H. (2019) Immunoglobulin heavy chains are sufficient to determine most B cell clonal relationships. *bioRxiv*, 665760.
